# Effects of Social Media Use on Connectivity and Emotions During Pandemic-Induced School Closures: Qualitative Interview Study Among Adolescents

**DOI:** 10.2196/37711

**Published:** 2023-02-23

**Authors:** Elisa Liang, Emily R Kutok, Rochelle K Rosen, Taylor A Burke, Megan L Ranney

**Affiliations:** 1 Brown-Lifespan Center for Digital Health Providence, RI United States; 2 Lynch School of Education and Human Development Boston College Chestnut Hill, MA United States; 3 Department of Psychiatry and Human Behavior Alpert Medical School of Brown University Providence, RI United States; 4 Department of Behavioral and Social Science Brown University School of Public Health Providence, RI United States; 5 Center for Behavioral and Preventive Medicine The Miriam Hospital Providence, RI United States; 6 Department of Psychiatry Massachusetts General Hospital Boston, MA United States; 7 Department of Psychiatry Harvard Medical School Boston, MA United States; 8 Department of Emergency Medicine Alpert Medical School of Brown University Providence, RI United States; 9 Department of Emergency Medicine Rhode Island Hospital Providence, RI United States

**Keywords:** social media, adolescents, COVID-19, emotions, connectivity

## Abstract

**Background:**

The COVID-19 pandemic provided a unique opportunity to examine social media and technology use during a time in which technology served as adolescents’ primary form of socialization. The literature is mixed regarding how increased screen time during this period affected adolescent mental health and well-being. The mechanisms by which screen time use affected adolescent psychosocial outcomes are also unknown.

**Objective:**

We aimed to deepen our understanding of how social media and technology use, social connectivity, and emotional well-being intersected during pandemic-related school closures.

**Methods:**

English-speaking adolescents aged 13 to 17 years were recruited on Instagram for a brief screening survey; 39 participants were purposefully selected to complete a semistructured interview regarding their social media and technology use during the pandemic. Interview summaries were abstracted from recordings, and deductive codes were created for the primary question stems. These codes were subsequently reviewed for the main themes.

**Results:**

The main themes were as follows: adolescent social media and technology use during school closures usually allowed for more and easier social connectivity, but the amount and relative ease of connectivity differed according to purpose and type of use. Emotions, particularly those of stress and happiness, were connected to whether adolescents actively or passively engaged with social media and technology.

**Conclusions:**

Our results suggest a nuanced relationship among social media and technology use, adolescent social support, and emotional well-being, including during the pandemic. Specifically, how adolescents use or engage with web-based platforms greatly influences their ability to connect with others and their feelings of stress and happiness. In the context of the COVID-19 pandemic and as technology in general remains at the core of the adolescent experience, future research should continue to examine how adolescents navigate and use web-based spaces in beneficial and harmful ways. This will inform education and interventions that foster healthy social media and technological habits.

## Introduction

### Background

Social media has become a ubiquitous part of adolescent lives. In a recent survey, 95% of adolescents in the United States reported the use of at least 1 social media platform and 97% of adolescents reported being on the web daily [1]. The percentage of adolescents who say they use the internet almost constantly has doubled in the past few years from 24% in 2014 to 2015 to 46% in 2022. This increasingly prevalent internet-based environment not only provides convenient ways for adolescents to connect and network with peers but also influences adolescents’ mental and emotional health. A national survey assessing youth risk behavior indicated a 40% increase in adolescents who reported persistent feelings of sadness or hopelessness from 2009 to 2019 [2]. As the rates of social media use and adolescent mental illness have escalated over the past decade, so have empirical investigations on the positive and negative effects of social media use on adolescent mental health and well-being. Social media may improve adolescent well-being by increased social support and feelings of connectedness [3,4]. It may also worsen adolescent well-being via increased depressive and anxiety symptoms [3,5]. Existing quantitative and qualitative research on adolescent social media use and well-being is limited in that it targets specific platforms, forms of social media use, or outcome variables rather than exploring a more holistic narrative of adolescents’ use of social media and technology. For example, numerous studies have examined the effects of passive Facebook use on well-being [6,7] and others have investigated how Facebook and Instagram were used to cope specifically with feelings of loneliness [8,9]; however, research exploring how complex web-based platforms may simultaneously provide positive and negative experiences is sparse.

A more nuanced examination of the sometimes-conflicting effects of social media on adolescent mental and emotional health is particularly important in light of the recent and ongoing COVID-19 pandemic. Increases in social media and technology use, increases in depressive and anxiety symptoms, and decreases in in-person support and interactions have been observed during pandemic-related school closures [10,11]. However, as in prepandemic studies, the relationship between social media use and mental well-being may be complicated. For example, 1 recent study found that higher total screen time use by adolescents during the pandemic was associated with poorer mental health and greater perceived stress, whereas lower screen time was associated with more social support and coping behaviors [12]. Other studies, in contrast, have found that young adults’ use of social media to express emotions was related to positive mental well-being during the pandemic and that organizing digital gatherings and reporting stronger web-based support were related to greater well-being and feelings of positive affect [13,14]. Our own work suggests that the prepandemic mental health effects of social media may be moderated by adolescents’ purpose for social media use such that adolescents who use social media for coping purposes may experience a protective effect from screen use compared with the negative correlation between increased screen use and well-being found in adolescents who infrequently use social media for coping purposes [10]. Other factors that may influence the relationship between adolescent social media use and psychosocial outcomes during the pandemic are social support and upward social comparison [15].

In others’ work, different media-related coping strategies were associated with different indicators of well-being in young adults during the pandemic. Young adults who used social media to joke about or reframe pandemic-related situations reported more positive affect and higher mental health scores than those who used social media for escapist and avoidant coping (defined as using social media to seek distraction from frustration, stress, or anxiety owing to the pandemic) [16]. In another study, humorous coping via social media (ie, watching funny videos alone or with others) was found to be associated with greater happiness among adolescents during the lockdown [17]. By definition, humorous coping may also qualify as avoidant coping; if so, these results contrast with the previous study and suggest that some forms of avoidant media coping may have beneficial effects. In support of this, some conceptualizations of escapist media used as a form of emotion-focused avoidance coping suggest that it may have positive short-term effects on well-being by temporarily reducing stress and anxiety [18]. More research is needed to better define the purposes for which adolescents use social media during the pandemic and its related effects on emotions and well-being to further distinguish and define potential adaptive and maladaptive social media coping strategies.

Qualitative research on social media use during school closures—and specifically the types of social media use that affect mental health—could help address this gap in knowledge and generate new hypotheses about both impact and potential interventions to enhance adolescents’ mental health in the context of the COVID-19 pandemic. A qualitative approach allows the generation of novel themes that can provide a framework for future inquiries [19]. Qualitative data may also provide insight into the nuances of how and why specific types of social media and technology use affect adolescent emotional well-being. Finally, qualitative evaluation of individual adolescents’ perspectives on the relationship between social media use and their emotions may drive future investigations of specific aspects of social media related to negative and positive affect.

### Objectives

This analysis was a component of a larger project examining adolescents’ mental health in the United States and changing technology use during the COVID-19 pandemic [10]. The goal of this analysis was to explore how adolescents’ interactions with social media and technology changed during school closures and how different types of social media use and purposes for social media use (eg, connecting with friends, scrolling through posts, etc) influenced perceived well-being. In this study, “school closures” refers to the time when students were not able to attend school in-person. We incorporated quantitative self-report measures on adolescents’ psychosocial outcomes (ie, depressive and anxiety symptoms and perceived social support) to contextualize our qualitative findings.

## Methods

### Recruitment

From September 23 to December 16, 2020, we used targeted Instagram advertisements to recruit adolescents (13-17 years of age, English speaking, and residing in the United States) to complete a brief screening survey administered using REDCap (Research Electronic Data Capture; Vanderbilt University) [20]; this screening survey confirmed basic demographics and assessed social media use and general well-being over the past 7 days [10]. All participants who completed the screening survey were asked to complete a web-based assent form and 6 comprehension questions that reviewed study protocols. The selection process for a subsequent qualitative interview began with the subsample of all adolescents who completed the assent process and was then refined in an effort to maintain purposeful representation of demographic subgroups (ie, representation across regions of the United States, race, gender, and age). Selected adolescents were phoned to confirm contact information, and parental consent was obtained. All participants interviewed completed a self-report survey using REDCap as well as a semistructured interview.

### Ethics Approval

This study was approved by the Rhode Island Hospital Institutional Review Board (#883341).

### Measures

#### Semistructured Interviews

A trained research assistant with a background in psychology conducted semistructured interviews with all consenting participants via a video call–based platform (Zoom). Participants were asked open-ended questions regarding general technology and social media use, how COVID-19 changed their technology or social media use, and the role of technology in their emotions (Multimedia Appendix 1). The interviews lasted between 15 and 51 minutes (average length 31, SD 8.85 minutes) and were digitally recorded. Qualitative debriefs (a brief review of the interview process, quality, and key data) were written after each interview to ensure quality and consistency. Interviews were continued until saturation of content was reached based on a review of the interviewer’s qualitative debriefs and discussion at team meetings.

#### Demographics

To measure age, race, ethnicity, and socioeconomic status, select self-report items from the National Study for Adolescent Health were included in the screening survey [21]. Gender was measured using 1 item from the Gender Identity in US Surveillance Group [22]. Sexual orientation was measured using 1 item from the Network for LGBT Health Equity at the Fenway Institute [23].

#### Mental Well-being and Social Support

The Patient-Reported Outcomes Measurement Information System (PROMIS)–Depression (PROMIS-depression) is an 8-item questionnaire that assesses depression symptom severity in the past 7 days [24]. The PROMIS-anxiety is an 8-item questionnaire that assesses anxiety symptom severity over the past 7 days [24]. Response options for both PROMIS scales were given on a 5-point Likert scale ranging from “1=at no time” to “5=all of the time.” The Multidimensional Scale of Perceived Social Support (MSPSS) was used to assess overall perceived social support from friends, family, and significant others [25-27]. The response options were on a 7-point Likert scale ranging from “1=very strongly disagree” to “4=half and half” to “7=very strongly agree.” As per standard practice, the PROMIS scale and MSPSS were scored as sums. We interpreted our summed scores according to the score translation table developed for these short forms [24], with raw scores of 32 to 40 indicating severe depressive and anxiety symptoms. For the MSPSS, we adhered to traditional cutoffs, with scores of 12 to 35 indicating low, 36 to 60 indicating medium, and 61 to 84 indicating high perceived social support.

### Analysis

Semistructured interview summaries were abstracted directly from the recordings by a qualitatively trained PhD student who listened to the audio recordings and noted the participant answers. Quotes were transcribed verbatim directly into the summaries. All summaries were read by a team of 3 research assistants and the interviewer to verify accuracy and completeness; discrepancies were discussed and resolved. The agreed-upon summaries were then entered into the NVivo (QSR International) qualitative data analysis software [28]. Deductive codes were created based on question stems (Multimedia Appendix 2) and applied by the interviewer. Any discrepancies were discussed and resolved by a research assistant, the interviewer, and a coinvestigator. Codes in which adolescents discussed their changes in social media use, connectivity, and emotions were abstracted and reviewed in further detail by the senior coinvestigator and the study team for main themes; memos were written for each theme and shared with the research team, and inductive codes were applied to the data based on the discussion of the initial coding scheme. The themes were developed based on discussions among the entire team. Codes were also examined comparatively in NVivo according to participant groupings based on descriptive cutoffs for the quantitative self-report measures (described earlier) to identify any patterns among those who reported severe versus nonsevere depressive and anxiety symptoms and high versus medium perceived social support.

Descriptive statistics for the entire sample were calculated using SPSS (IBM Corp).

## Results

### Overview

Semistructured interviews were conducted with 39 adolescents aged 13 to 17 (mean 15.9) years; approximately half of the participants identified as women (49%), and most were non-Hispanic (85%; see Table 1 for the demographics of our study sample). Table 1 shows the demographics and quantitative scores of our study sample. To protect our participants, we did not disclose specific demographic information for the quotes used in this paper; in summary, we used 31 quotes from 19 different participants who reflect the diversity of our overall sample.

Qualitative themes were examined comparatively between those who reported high (31/39, 79%) versus medium (8/39, 21%) social support on the MSPSS (no participants reported low social support). Data were also compared between those who reported severe (6/39, 15%) versus nonsevere (33/39, 85%) depressive symptoms and severe (8/39, 21%) versus nonsevere (31/39, 79%) anxiety symptoms.

There were no observed differences in the themes based on social support or well-being.

We identified two overall themes related to technology and social media use during school closures: (1) perceived social connectivity and (2) emotions. Each is described in detail in subsequent sections. For theme 1, we found that connectivity had several subthemes, each related to the amount and relative ease of connectivity that adolescents experienced with their peers on the web. For theme 2, we found that emotions, particularly that of stress and happiness, were connected to whether adolescents actively or passively engaged with social media and technology.

**Table 1:** Demographics of study sample (N=39).

Demographic variables		Values, n (%)
**Self-reported gender**		
	Man	10 (26)
	Woman	19 (49)
	Nonbinary or transgender	8 (21)
	Prefer not to answer^a^	2 (5)
**Sexual orientation**		
	Straight	11 (28)
	Gay or lesbian	6 (15)
	Bisexual	10 (26)
	Other	12 (31)
**Ethnicity**		
	Hispanic	6 (15)
	Non-Hispanic	33 (85)
**Race**		
	Asian	6 (15)
	Black or African American	3 (8)
	White	26 (67)
	Mixed	4 (10)

^a^One participant chose not to disclose their gender.

### Theme 1: The Effect of Technology and Social Media Use on Perceived Social Connectivity During School Closures

#### Overview

Overall, participants indicated that their use of technology and social media increased during school closures owing to the pandemic. The reasons provided for this increase included taking web-based classes, maintaining social relationships, and keeping oneself entertained during school closures. Staying in touch with friends and peers emerged as a primary reason for increased social media and technology use in all 39 interviews. Participants described how social media both facilitated and hindered their ability to connect with others during this period of school closure.

#### More Social Connectivity

Most participants reported that social media and technology helped them stay connected with others during school closures in the COVID-19 pandemic. Most participants said they stayed in touch with their core group of friends using social media and that their number of interactions increased. Many participants said that they also used social media to create new friendships during this period. There was considerable overlap between these 2 groups; 10 participants reported that they both spoke to the same friends and made new friends on the web. A few participants said that because of technology, they became distant from formerly close friends while becoming closer to people they had not known well before. Many participants reported meeting new web-based friends through existing in-person friends:

I used to just hang out with the people who sat with me at lunch, but now it’s friends of friends of friends

on Discord

Discord is an audio-, text-, and video-based communication application whose use originated with gamers [29]; it is particularly known for its voice chat and screensharing features that allow groups of users to speak and view content together in a real-time setting.

#### Easier Social Connectivity

Social media and technology were described as making communication easier according to approximately a quarter of the participants. Some highlighted that it was less intimidating to approach people on the web than in person, for example:

It’s not as scary as interacting in person...before, some people may seem too intimidating to approach, but now it’s like you don’t really know how anyone looks like or sounds like so it’s not really much of a fear.

Others mentioned how social media made it easier to connect with people from around the world:

[Social media] allows me personally to talk to friends I may never have met previously who live all over the globe, which is quite cool.

A few adolescents pointed out that when it comes to communicating with others via technology, emotions can be more accurately represented through GIFs, videos, and emojis than through words. Others talked about how being on the web allowed them to act more like their true selves without fear of judgment from others:

I’m really bad at talking to people in real life but online it’s so much easier and I’m really extroverted online but really introverted in person...so it just makes it a lot easier to make friends and talk to people and share stuff.

Although these advantages of technology may have become particularly salient during the pandemic, the participants also spoke about them generally, implying that they were true before the pandemic as well.

Notably, Discord was mentioned by most participants as a platform that facilitated web-based connectivity during school closures in the COVID-19 pandemic; 8 participants said that they first started using Discord during the pandemic. Of the participants who used Discord before school closures, most reported more frequent use during the pandemic compared with before school closures. Ten participants explicitly stated that they used Discord to game with friends; the others used it to voice chat with friends, video call friends, or message friends.

#### Hindered Social Connectivity

More than half of our participants talked about the difficulties in interpreting web-based communications during school closures (which many referred to as “quarantine”). For example, adolescents mentioned that being unable to see their peers made it harder to read the other person’s feelings and intentions through text messages. They also mentioned that they missed the cues from physical touch:

Just that like when it is texting...I can’t see what their facial expressions are...it gets kinda confusing if they’re being sarcastic about something or not, or if they are joking and—I don’t know, it’s just nice to be near people...you know touch them on the shoulder or something and you can’t do that now.

Another participant spoke about how specifically the loss of familiarity with their peers’ personalities made it more difficult to interpret tone and intentions over text messages:

Before [quarantine] I would see people during class...so I would know what their personality is like, but I don’t know how they’ve changed during quarantine, so I don’t know how they’re thinking or if what they’re saying lines up with my idea of them that I would see in-person.

Many participants spoke about how communicating over social media and technology lacked emotional intimacy and sincerity overall, especially without in-person interactions to supplement:

I feel like on social media and stuff people are less real, it feels a little more disingenuous. Like I totally get it, but it’s harder. It feels less real.

#### Less Social Connectivity

Approximately a quarter of the participants described how social media and technology fell short when it came to reaching others during the pandemic. Five talked about difficulties getting in touch with others on the web owing to peers failing to or taking a while to respond:

There can be an element of stress to it, waiting for a text or “why isn’t this friend texting me back.”

Compared with in-person conversations, web-based conversations can also be suddenly dropped by 1 party without explanation:

It can be a little unsatisfying if you actually want to have a conversation with a person and then you send a couple of texts back and forth and they stop responding and you’re like “I don’t know if it’s that they don’t want to talk to me or if they just had to take their dog for a walk or something” I don’t know.

Another handful of participants elaborated on situations where outdated technology or poor internet connections created challenges:

I started using Twitter...two months ago but that’s mostly to talk with my friends...because some of their internet is really bad and they can’t use Discord because it requires good internet.

Furthermore, 1 participant described how they became more selective with who they chose to stay in touch with after-school closures:

So I stopped using it [Snapchat] also because we’re not seeing people in person, I don’t know if this makes sense, I don’t feel like I’m obligated...some people I follow on Snap that I don’t know as well, now that I don’t see them it’s like I don’t really need to talk to them.

Similarly, another participant spoke about becoming generally disconnected from peers during this time because of the lack of in-person contact:

I’m barely talking to anyone. After we got out for school, I kind of just accidentally cut everyone off and no one heard from me...because I didn’t really talk to anyone outside of school. It was mostly just an in-person thing. So now that we weren’t going to school it’s like oh, sorry I didn’t talk to you.

### Theme 2: The Effect of Technology and Social Media Use on Emotions During School Closures

#### Overview

Participants were asked specifically about whether social media and technology use made them feel stressed or happy and how these emotions may have changed after school closures. Most participants denied experiencing increased stress from screen use during school closures. The remaining participants stated that they felt more stressed after school closures owing to constantly being on social media, especially owing to engaging with pandemic-related content. Although only a little more than half of the participants directly addressed whether their screen-use–related happiness changed after school closures, most of those participants claimed that their happiness stayed the same, whereas a handful reported feeling happier and only 3 reported feeling less happy.

Participants were also asked separately about instances when they felt happy and stressed owing to social media and technology use. From their responses, inductive themes surfaced regarding active and passive social media and technology engagement. As these emerged, we defined “active engagement” as per others’ work [30] as creating posts on platforms, directly messaging others, or engaging with others in web-based public spaces, such as comments sections, videogame servers, etc; for example, “I post pictures of cats because I like to make people feel happy.” We defined “passive engagement” as browsing through posts, watching videos, and engaging in any web-based content alone, exemplified by “I see people who are working out, getting really fit and that really motivates me...” Each of the following subthemes about stress and happiness owing to screen use are organized into subsections for active and passive engagement.

#### Sources of Stress—Active Versus Passive Engagement

##### Active Engagement

Negative interactions with others on technology and social media were a common source of stress and being upset for participants during school closures, with over one-third of participants describing such cases. This included stress from receiving upsetting messages or comments while gaming, friend group drama, unpleasant conversations in group chats, and difficulty communicating with others over SMS text messaging. Although some of these experiences predated the pandemic, participants indicated that their frequency or tenor had increased or changed during school closures. Commonly described sources of conflict included politics; the pandemic; and lesbian, gay, bisexual, transgender, queer topics. Four participants explicitly underlined the stress from expressing differences on social media. One of them said that active engagement of social media for political purposes increased stress during the pandemic:

Just because I have been posting more political stuff so then people refute my political stuff and then I rebut that and then it’s a thing.

Another participant described an unpleasant time when they tried to clarify misinformation but were shut down:

During the BLM protests, there was a thing where people were...spreading a little misinformation...and I know that that isn’t a thing, that’s not a law, so I disagreed with them, but I ended up being downvoted a ton, so for disagreeing with facts, I was kind of censored.

A few participants recalled receiving mean or upsetting messages from other social media users. Specifically, 1 participant mentioned that they were called “*ugly*” in typed comments from strangers on the web, and another said that they received mean text messages from friends of friends that they did not know well. Three participants explicitly spoke about being cyberbullied during school closures by people who had disliked their posts and moving or removing public social media accounts as a result. One of these adolescents felt that they were being cyberbullied more during the pandemic than before school closures; this participant said that cyberbullying in general has increased during the pandemic because everyone is on the web and “people are finding reasons to get upset over something very minor.”

##### Passive Engagement

Negative passive engagement, which involves viewing but not participating in negative content or interactions on the web, was reported as a source of stress and upset for a large majority of participants. Participants described, for example, how passively observing negative posts or web-based arguments about politics; pandemic restrictions; and lesbian, gay, bisexual, transgender, queer topics generated more frequent stress than direct involvement in arguments or conflict:

I’ve been seeing a lot of not great stuff. I’m trans, so I’ve been seeing stuff about hate of trans people. That makes me uncomfortable. And Black Lives Matter stuff is stressful.

One participant observed that especially during school closures, passive engagement with social media increased exposure to conflict:

In real life, you might get bad news that is more connected to you, with social media, depending on what you’re looking at or connected to, you’ll get bad news of everything in the world. And that can lead to heavy fatigue if you’re not careful about it.

Other sources of passive negative engagement described by participants to generate stress included witnessing incidents of cyberbullying, often involving racist or homophobic statements and political disagreements. Many participants observed that cyberbullying occurred more often during school closures owing to increased time spent on the web and lack of in-person contact with the survivors:

They don’t have to be face to face with that person, so they send whatever they want. People aren’t scared of what they say behind the screen.

One participant (bystander) provided a specific example of this occurring to 1 survivor:

It [the bullying] definitely got more like aggressive now that they weren’t seeing her [the victim] in-person every day.

Web-based conflict and cyberbullying were also described to become more severe during the pandemic owing to the politically charged atmosphere and clashing of passionate and controversial opinions:

Before [the pandemic] it was just kinda making fun of people for maybe how they looked or if they did something stupid, but now it’s about much more serious topics, presidential stuff, racism, xenophobia, homophobia, stuff like that. It’s much more serious and people...feel much more passionately about it than they do about calling somebody stupid or saying that their dress is ugly.

In particular, 1 participant felt upset when they saw cyberbullying related to living circumstances affected by the pandemic:

A lot of people are just like shaming people and judging people for what they have like how their families are surviving this. Because like a lot of people are out of their jobs, and people are like oh my gosh why can’t you get another job. That’s...not how it works.

However, some participants also felt that more people were speaking up against cyberbullying than before the pandemic:

Now more than ever, people who used to not say anything are getting up and saying what they think. After COVID, they have realized, maybe it’s better to stand up and say what they need to say. I feel a lot of new people are sharing their thoughts about conflict.

In addition, many participants spoke about feeling stress from comparing themselves to others on the web, including comparisons to the amount of engagement that others were receiving. This source of passive stress reportedly increased during school closures as well. One participant explained as follows:

I feel like I followed more people since the pandemic started so I feel like I should get more likes and more comments and stuff. It kind of brings down your self-esteem too, like I base my worth on how my Instagram is.

Some participants reported being upset when they juxtaposed their pandemic lives with others, such as 1 adolescent who said:

Sometimes it kind of makes me feel down because I see people out doing stuff and I’m like I wish I could be doing that.

Finally, it was also stressful for 1 participant to see peers violating COVID-19 pandemic stay-at-home mandates:

I’m trying to be safe and then there are some people who just don’t care. It’s upsetting.

#### Sources of Happiness—Active Versus Passive Engagement

##### Active Engagement

The types of active web-based engagement associated with self-described happiness or enjoyment during school closures included communicating, playing games, and sharing art with friends. For example, in reference to technology and social media, 1 adolescent said:

I enjoy to be on it now [more than before], like I follow a bunch of artists so I like it when I see them do artwork and like sharing...[their posts] with my friends.

One participant mentioned that seeing their peers’ social media posts made them feel happier now compared with before the pandemic:

I think I might actually like looking at people’s pictures better now, because I didn’t look at them that much before, like at all...it just gives me a way I can talk to people and even see how they’re doing.

Overall, many participants cited social connection as their reason for experiencing happiness from social media use, particularly in the context of school closures:

Seeing that my friends are doing okay, seeing that my friends are doing well and fine, and getting to wish them happy birthdays and stuff, like outside of in person, it makes me happy.

This aligns with a general sentiment of appreciation toward social media and technology from participants who realized how crucial these platforms were for communication during the pandemic:

I didn’t really acknowledge it [happiness from social media use] as much before COVID, but when I think about it I’m like what would I be doing in COVID without this.

Over half of the participants expressed that they felt enjoyment or happiness from active technology and social media use during school closures.

##### Passive Engagement

Types of passive engagement associated with self-described happiness or enjoyment primarily involved viewing posts with positive or entertaining content (cat videos, artwork, inspiring messages, etc).

A handful of participants elaborated that they specifically used technology or social media passively when they were feeling down:

If I’m feeling really kind of upset and I just want to distract myself, if I just start watching cat videos or something, I tend to feel a bit better.

Notably, this participant described this feeling to be distinct from that of happiness (“I wouldn’t say it makes me feel happy, but I would say it makes me feel less bad”).

Especially during school closures, participants seemed to rely on social media use as a source of support or coping strategy for pandemic-related stressors. One participant spoke about social media being a motivating force for them to get out of bed and be productive each morning. Similarly, another participant explained how social media was helpful when present issues became too overwhelming:

Sometimes it is good because if I need a break from reality, I can just connect with a page I really like, just look at their photos and that kind of reminds me of when stuff was open or when things were normal.

Furthermore, a few participants mentioned that they purposefully customized their social media feed to only show content that made them feel happy. This was described as a particularly useful skill during the pandemic when social media was overrun with controversial discourse:

I realized you can basically remove people you don’t want to hear anymore, so I began unfollowing people, I began deleting apps, and I began following people who kind of brought me happiness, so now whenever I go on social media, it’s never like “oh no, I don’t want to go on social media, why??” but now it’s just like “oh look, another good story.”

Overall, passively engaging on social media was related to positive emotions in over one-third of the participants.

## Discussion

### Principal Findings

This first-of-its-kind qualitative analysis explores adolescents’ own perspectives on the value and pitfalls of social media and technology use during the COVID-19 pandemic. Although many findings mirrored prepandemic challenges, we identified several novel and important themes in the context of the COVID-19 pandemic. We identified the following: (1) new use of old technologies (ie, Discord), (2) new sources of stress, (3) novel benefits of active and passive social media use, (4) changes in web-based social circles and social experiences, (5) persisting limitations of social media use, and (6) changes in cyberbullying and bystander behaviors. Surprisingly, we found that these challenges and opportunities cut across our demographically and experientially varied group of participants, with no major thematic differences according to gender, age, perceived social support, and emotional well-being. Although increased time on social media during the COVID-19 pandemic was previously associated with lower mood and poorer mental health outcomes in adolescents [12], our analysis provides insights into how the purpose and type of technology use influences both positive and negative outcomes.

Although much research discussion of adolescent technology use has focused on social media platforms such as TikTok, Instagram, and Snapchat [31], Discord emerged as being among the most used platforms for our participants. Discord is a web-based application that allows messaging, voice chat or video calls, and playing cooperative video games. Previous research on Discord has highlighted its use for multiplayer video games and its potential for subversion by White nationalists [32,33]. Others’ work has evaluated Discord’s potential to improve distance learning owing to its attractive user interface, completeness of features, and ease of maintaining social links between students and professors [34,35]. Our work confirms this potentially positive value of Discord for adolescents, particularly during the pandemic era. For example, the ability to combine voice and text chat may better emulate the feeling of in-person conversations without the associated pressures of being in-person [29]. Our findings therefore raise new insights about the value of nonvideo gaming uses of Discord for social connection; both observational and interventional research is needed. Finally, we urge researchers to include Discord as an identified social media platform in future survey research.

We identified novel web-based stressors during the pandemic, namely observing others’ noncompliance with COVID-19 pandemic stay-at-home mandates and observing or participating in polarizing web-based discourses surrounding the pandemic, politics, and other controversial events during the time participants were interviewed. Although prepandemic research on active social media use suggests that active engagement is typically associated with positive well-being outcomes [7,36], emerging research suggests that active engagement with COVID-19 social media content during the pandemic is related to higher anxiety [37]. Illustratively, in our study, actively participating in web-based discussions about current events such as the Black Lives Matter protests and distancing regulations served as sources of stress that adolescents had not experienced previously. During the pandemic, social media platforms became the primary medium for the widespread dissemination of news, information (and misinformation), and political propaganda [38], which kept adolescents informed about current events and perspectives and provided endless fuel for discussion and debate. We suggest that the purpose behind active social media use (social connection vs political discourse) may influence its effects on well-being. Future work should quantitatively explore the relative influence of health or political mis- and disinformation and provide guidance to youth on how to proactively identify and manage potential political and social conflicts on the web.

The value of active social media engagement in this analysis parallels that of prepandemic research [7,36] such that participants reported that using social media to connect with others led them to experience positive affect. However, active engagement in social media during school closures was identified as qualitatively different from that before school closures. For example, some participants mentioned that messaging and sharing content with friends on social media was more enjoyable than in prepandemic times. Overall, social connection with friends emerged as the predominant source of happiness from active screen use during the pandemic. Our analysis also aligns with prepandemic research reporting that passive social media use is more strongly correlated with negative well-being than active use [6,7,14], particularly around the impact of social comparison [15,39]. However, participants also mentioned that passively using social media as a distraction served a functional purpose by making them feel better when they were overwhelmed by pandemic-related stressors. One adolescent even used the phrase “a break from reality” to describe the reason for their screen use. In discussing how passive screen use improved their affect, participants more frequently recalled times when passive use relieved negative feelings rather than explicitly instilling positive feelings. These reports support previous work suggesting that avoidance coping using social media may sometimes be beneficial by allowing adolescents to engage with preferred content and, in turn, temporarily mitigate stress and other negative emotions [18].

Another prominent theme identified in our analysis was how technology both enhanced and hindered adolescents’ ability to socially connect with friends and peers during school closures. Web-based platforms being the sole method of communication introduced unique challenges described by participants. Prepandemic research highlights the ways in which social media allows youth to create and maintain larger social circles [40,41]. Although this was still true for many of our participants during school closures, this analysis found that social media was sometimes deliberately or unintentionally used by adolescents to limit social circles during this period. Others reported naturally losing contact with peers over time. Before the pandemic, adolescents ascribed digitally disconnecting from others to web-based drama or bullying [42]; however, digital disconnect during the pandemic was attributed more simply to the lack of in-person contact to propel or supplement peer interactions. Nonetheless, our study sample reported a relatively high perceived social support level despite being during school closures, implying that feelings of social support can be maintained through social media in the absence of in-person interaction. We were unable to tease out perceived social support among adolescents who connected primarily with friends they already knew in person versus adolescents who made new friends during this period. Future studies should examine how adolescents’ perceptions and acceptance of relationships that are only based on the web have changed over the course of the pandemic and how different forms of social support may influence the effects of social media use on well-being outcomes.

Although school closures enhanced some positive aspects of technology use for connectivity, they also exacerbated preexisting negative aspects of web-based communications such as the potential to misunderstand and misinterpret text messages [43]. Despite most participants seeming to acknowledge or even prefer the convenience of messaging others (compared with talking in person), they also reported missing the ability to complement text- or image-based communication with in-person interactions and physical touch. Adolescents previously acknowledged the superficiality of web-based communication in prepandemic times, stating that such communication lacked emotional depth [43], but school closures and the complete absence of in-person contact seemed to exacerbate the impersonal feeling of digital messages reported by many participants. Evidently, there are crucial aspects of connectivity that only web-based interactions cannot fulfill—an important consideration for schools and youth-based organizations as the world continues to digitalize.

Exposure to and experiences of web-based bullying also changed during the pandemic [44,45]. Participants described an increase in the frequency of bullying owing to increased screen time as well as new forms of bullying around pandemic-related behaviors. On the positive side, participants also mentioned an increase in their peers’ use of prosocial bystander behaviors. Prepandemic research on bystander intervention in cyberbullying found that increased cognitive empathy (ie, mental perspective taking) and interethnic contact were related to prosocial bystander behaviors among adolescents [46,47]. The shared struggle of pandemic-related school closures and greater exposure to news and web-based slander targeting minority groups during this time may have increased overall empathy among adolescents and encouraged more of them to speak out against perceived injustices.

There were no differences in the patterns of themes based on participants’ age, gender, mental well-being, or perceived social support. The overall study sample reported high perceived social support and low depressive and anxiety symptoms, indicating that adolescents’ expressed stressors and benefits of using social media and technology during the pandemic did not depend on their personal well-being. The lack of thematic differences in our varied sample suggests that the aspects of social media and technology use that adolescents found challenging and helpful during the pandemic may be universal. Active and passive screen use were also associated with stress and happiness in a similar manner across groups, implying that it may be appropriate and effective to standardize education on the pros and cons of social media and technology use for all adolescents. However, it remains true that there were a variety of narratives describing unique experiences with social media and technology use during the pandemic. This suggests that there may be other variables influencing adolescents’ connectivity and feelings of stress and happiness, such as their prepandemic familiarity or comfort with web-based communication or time spent using social media and technology during the pandemic.

### Limitations

Although we interviewed participants until we reached content saturation, our small study sample may have contributed to the lack of observed differences in interview themes across participants. Furthermore, the nature of a qualitative analysis prevents it from establishing any causality or generalizability between themes and adolescents’ self-reported well-being. Although efforts were made to recruit a nationally representative sample of adolescents across multiple levels of identity, the subset of participants who consented to and fully completed the interview was less diverse in terms of racial and ethnic identity; however, we were able to achieve greater representation of sexual and gender identities. The study sample was also recruited solely from Instagram, so it is possible that these participants differed from those who do not use Instagram. Although these semistructured qualitative interviews were guided by a written agenda, the nature of qualitative research is such that comments and provided details varied based on participants’ individual experiences. This analysis was conducted from written summaries with verbatim quotes but not from full transcripts. Summaries, however, clearly noted when participants had no comments on particular open-ended questions and are appropriate alternatives to transcription-based analysis [48]. This analysis provides a preliminary framework of adolescents’ social media use during the pandemic on which further research should continue to refine and build on.

### Conclusions

Overall, this qualitative analysis on changes in adolescent social media and technology use during pandemic-related school closures demonstrates that the effects of active and passive engagement on social connectivity and emotions during this time were complex and variable, depending on the purpose for which they were used. Although evidently useful for maintaining and facilitating social connections among peers, especially during a time when in-person contact was minimal, it was also clear that there were areas where social media and technology could not replace in-person communication. Furthermore, although active web-based engagement may have been more frequently associated with happiness during school closures compared with passive engagement, it was also a source of stress for participants who participated in polarizing web-based discourse. Although passive web-based engagement was more frequently related to stress during this time, adolescents also demonstrated that they could exert control over their passive social media use to make it more beneficial and uplifting. Researchers should seek to develop and disseminate strategies to guide adolescents on how to personalize their social media experiences to be more positive rather than negative. Consideration of how patterns of use and related outcomes shift as youth return to in-person school—and how to enhance positive impacts in case of short-term, future school closures—is also needed. Ultimately, social media and technology research and education for adolescents remains essential given the prominence of web-based environments in their social lives.

## Appendix

Multimedia Appendix 1 Abbreviated semistructured interview question guide.

Multimedia Appendix 2 Deductive codes corresponding to the abbreviated semistructured interview question guide.

## Abbreviations

MSPSS: Multidimensional Scale of Perceived Social Support
PROMIS: Patient-Reported Outcomes Measurement Information System
REDCap: Research Electronic Data Capture
