# Supplementary material for: Effects of Social Media Use on Connectivity and Emotions During Pandemic-Induced School Closures: Qualitative Interview Study Among Adolescents
Source: JMIR Ment Health. 2023 Feb 23;10:e37711. doi: 10.2196/37711 (PMC9953983; doi:10.2196/37711)
Supplement: Multimedia Appendix 1 [file mental_v10i1e37711_app1.docx]

**Multimedia Appendix 1. Abbreviated semistructured interview question guide.**

**General technology use**

1. To start, I want to talk about what technology / social media you use? One of the things we want to understand is if your technology use has changed. So, when you answer this question, think about your technology / social media use before the COVID-19 Pandemic.
2. Have there been any major life changes e.g., moving, losing a job, starting a job, starting a new school that are not specifically COVID-19 Related?

**How COVID-19 has changed their technology/social media use**

1. Talk to me about a snapshot of what happened to your school this spring, and what it looks like now?
2. How else is your social media use different than it was before the COVID-19 pandemic?
3. How do/did you connect with your friends during the social distancing/stay at home?
4. Did your parents/guardians put rules into place or encourage you to stay on/off your phone, gaming console, other technology?
5. Are there any other offline activities that you are now doing via technology / social media that you weren’t doing before COVID-19 and how has that changed?

**Role of Technology in Emotions**

1. What are the good and not-so-good parts of connecting with people by technology / social media?
2. What role does technology / social media play on your emotions? How has that changed during COVID-19?
   1. Does it make you feel happy? How so?
   2. Does it make you feel stressed? How so?
3. How about bullying or violence online? Has that changed during COVID?
4. Has COVID affected romantic relationships or hookups either in person or online?
5. Are there things you wish you’d known 6 months ago about technology during COVID?
