# Supplementary material for: Effects of Social Media Use on Connectivity and Emotions During Pandemic-Induced School Closures: Qualitative Interview Study Among Adolescents
Source: JMIR Ment Health. 2023 Feb 23;10:e37711. doi: 10.2196/37711 (PMC9953983; doi:10.2196/37711)
Supplement: Multimedia Appendix 2 [file mental_v10i1e37711_app2.docx]

**Multimedia Appendix 2. Deductive codes corresponding to the abbreviated semistructured interview question guide.**

***SECTION B. GENERAL TECH USE (BEFORE COVID & IN GENERAL)***

- B.1.a. Social media platforms before COVID-19
- B.1.b. Time spent on Most Used Social Media Before COVID-19
- B.1.c. Definition of Technology use
- B.2. Major Life Changes Since COVID-19
- B.2.a. Effects on Technology Use
- B.2.b. Increases or Decreased in Social Media Use

***C. HOW COVID19 HAS CHANGED THEIR TECHNOLOGY/SOCIAL MEDIA USE***

- C.3. School in Spring vs. School Now
  - C.3.a Remote Status
  - C.3.b. Forms of Online Learning
    - C.3.b.i. Positives/Negatives of Technology Enabled Classes
  - C.3.c. Connections with Peers using Zoom/Social Media
- C.4. Changes in Social Media after COVID-19
  - C.4.a. Changes in Amount of Use
  - C.4.b. Changes in Timing of Day
  - C.4.c. Changes in Purpose of Use
- C.5. Connecting with Friends during COVID-19
  - C.5.a. Platforms/People Now
  - C.5.b. Different People Now
  - C.5.c. Different Platforms Now
  - C.5.d. Texting More or Less Now
  - C.5.e. Seeing People In-Person
  - C.5.f. Ways of Connecting Now
    - C.5.f.i. Apps in Use
    - C.5.f. ii. Devices in Use
    - C.5.f.iii. Differences from Before COVID-19
  - C.5.g. Zoom Fatigue
    - C.5.g.i. Limits on Screen Use
- C.6. Parent/Guardian Rules about Technology Use
  - C.6.a. Differences from Before COVID-19
- C.7. Offline Activities that are Now Online

***D. ROLE OF TECHNOLOGY ON EMOTIONS***

- D.8.a. Tech/Social Media Help Connections
- D.8.b. Tech/Social Media Make Connections Harder
- D.9. Role of Tech/Social Media on Emotions
  - D.9.a. Happiness from Tech/Social Media
  - D.9.b. Stress from Tech/Social Media
  - D.9.c. Upsetting interactions from Tech/Social Media
  - D.9.d. Technologies for Socialization Easier/Harder
  - D.9.e.  Examples of Favorite Social Media on Emotions
- D.10.  Bullying/Online Violence Changes
  - D.10.a. How has it Changed
  - D.10.b. Differences in Types of Conflict/People who engage in Conflict
  - D.10.c. Effects of Online Violence/Bullying on Self: More/Less than Before COVID-19
- D.11. Effects of COVID on Romantic Relationships
  - D.11.a. How has it Changed
  - D.11.b. Exploring More or less Romantically with Peers in Community
  - D.11.c. Exploring More or less Romantically with Online Only Adolescents
- D.12. Hindsight Knowledge about Technology Before COVID Started
  - D.12.a. Hindsight Knowledge about Technology Before COVID Started for Other Adolescents
  - D.12.b. Advice about Technology
  - D.12.c. Future Use Changes
